# Association between relative fat mass and osteoarthritis in American adults

**DOI:** 10.3389/fnut.2025.1610950

**Published:** 2025-06-18

**Authors:** Ziyuan Li, Tangchen Yin, Yijing Chen, Jiangsheng Huang, Yuanyue Jiang, Wei Deng

**Affiliations:** ^1^Department of Nursing, School of Health and Nursing, Wuxi Taihu University, Wuxi, China; ^2^Department of Pathology, Kunshan Hospital of Traditional Chinese Medicine, Suzhou, China

**Keywords:** RFM, NHANES, osteoarthritis, obesity, cross-sectional study

## Abstract

**Background:**

Relative fat mass (RFM) is a newly established anthropometric measurement that offers an alternative method for assessing body fat. Osteoarthritis (OA) is a widespread public health issue, with existing evidence identifying obesity as a notable risk factor for the OA development. This study aimed to examine the potential correlation between RFM and OA within a nationally representative population.

**Methods:**

This study employed data collected from the National Health and Nutrition Examination Survey (NHANES) from 2007 to 2018. Weighted logistic regression models were performed to assess the relationship between RFM and OA. Furthermore, the predictive efficacy of various adiposity indicators for OA was examined through receiver operating characteristic (ROC) curve analysis, allowing for comparisons of area under the curve (AUC) values.

**Results:**

The study cohort comprised 28,535 participants from the NHANES dataset. The analytical results indicated significant positive associations between RFM and OA. Stratified analyses revealed notable effect modifications based on age and diabetes status concerning the RFM-OA relationship. Comparative ROC analysis indicated RFM exhibited a superior capability for predicting OA prediction (AUC values: 0.646) compared with traditional obesity metrics.

**Conclusion:**

The findings demonstrated a significant positive correlation between RFM and OA, indicating that higher RFM levels are associated with increased risk of OA. ROC analyses reinforced the diagnostic value of RFM for OA, with its predictive performance exceeding that of conventional adiposity metrics.

## Introduction

Osteoarthritis (OA) is a prevalent musculoskeletal disorder, characterized by alterations in articular cartilage, subchondral bone remodeling, and osteophyte formation (1). Additionally, OA progression is characterized by concomitant synovitis and degenerative changes in periarticular tissues, including meniscal degeneration, tendinopathy, and fibrotic transformation of the infrapatellar fat pad (IFP). Studies have demonstrated progressive IFP volume loss and structural fibrosis in end-stage OA, establishing IFP remodeling as a hallmark pathological feature of advanced disease (2). It is estimated that over 590 million people worldwide are affected by OA, with its prevalence progressively increasing with age (3). Moreover, OA is a direct cause of substantial joint pain, leading to functional limitations and emerging as a primary contributor to disability (4, 5). Its significant individual and societal impacts are particularly pronounced among older adults (6). However, the pathogenesis of OA remains inadequately understood. Recent findings have demonstrated OA was closely associated with sociodemographic factors such as age, sex, and socioeconomic status, (7) as well as case-related factors including genetic susceptibility and synovial inflammation (8). Given its high prevalence and consequential effects, the identification of risk factors for OA is of paramount importance.

Obesity is a metabolic disorder characterized by an imbalance between caloric intake and expenditure, leading to excessive body fat deposition. Although the intricacies of the relationship between OA and obesity have not been fully clarified, prior studies have documented a significant correlation between knee osteoarthritis (KOA) and obesity (9). Excessive weight gain may further exacerbate the stress on the knee joint, leading to further joint degradation (10). Meanwhile, the function of adipose tissue should not be overlooked. During metabolic regulation, adipose tissue converts surplus energy into fatty acids and glycerol and releases adipokines (11, 12), thereby contributing to systemic chronic inflammation. Recent studies have illustrated that obesity promotes the production of adipokines, which subsequently contribute to the development of osteoarthritis (13, 14).

The Body mass index (BMI) remains the predominant metric for evaluating obesity, with a threshold of BMI ≥ 30 denoting obesity. Nevertheless, BMI exhibits certain limitations in accurately reflecting obesity, as it fails to differentiate effectively between muscle and fat mass (15). Individuals with identical BMI values can possess varying distributions of body fat (16). Recently, researchers have explored more precise anthropometric indicators of obesity, including the Body Shape Index, the Body Roundness Index, and Relative Fat Mass (RFM). RFM has emerged as a promising measure that provides a more precise estimation of total body fat percentage in contrast to conventional metrics. It is calculated using a straightforward formula based on height and waist circumference, making it a practical tool for both clinical application and research settings (17). RFM has demonstrated significant associations with various health conditions, including stroke (18), depression (19) and type 2 diabetes (20).

However, limited research has investigated the potential link between RFM and OA. Considering the accuracy of RFM in predicting health outcomes and the established correlation between OA and obesity, we hypothesized that RFM might also be associated with OA. This study utilized information obtained from the National Health and Nutrition Examination Survey (NHANES) database to assess the potential relationship between RFM and OA.

## Methods

### Study design and study population

**Figure 1:**
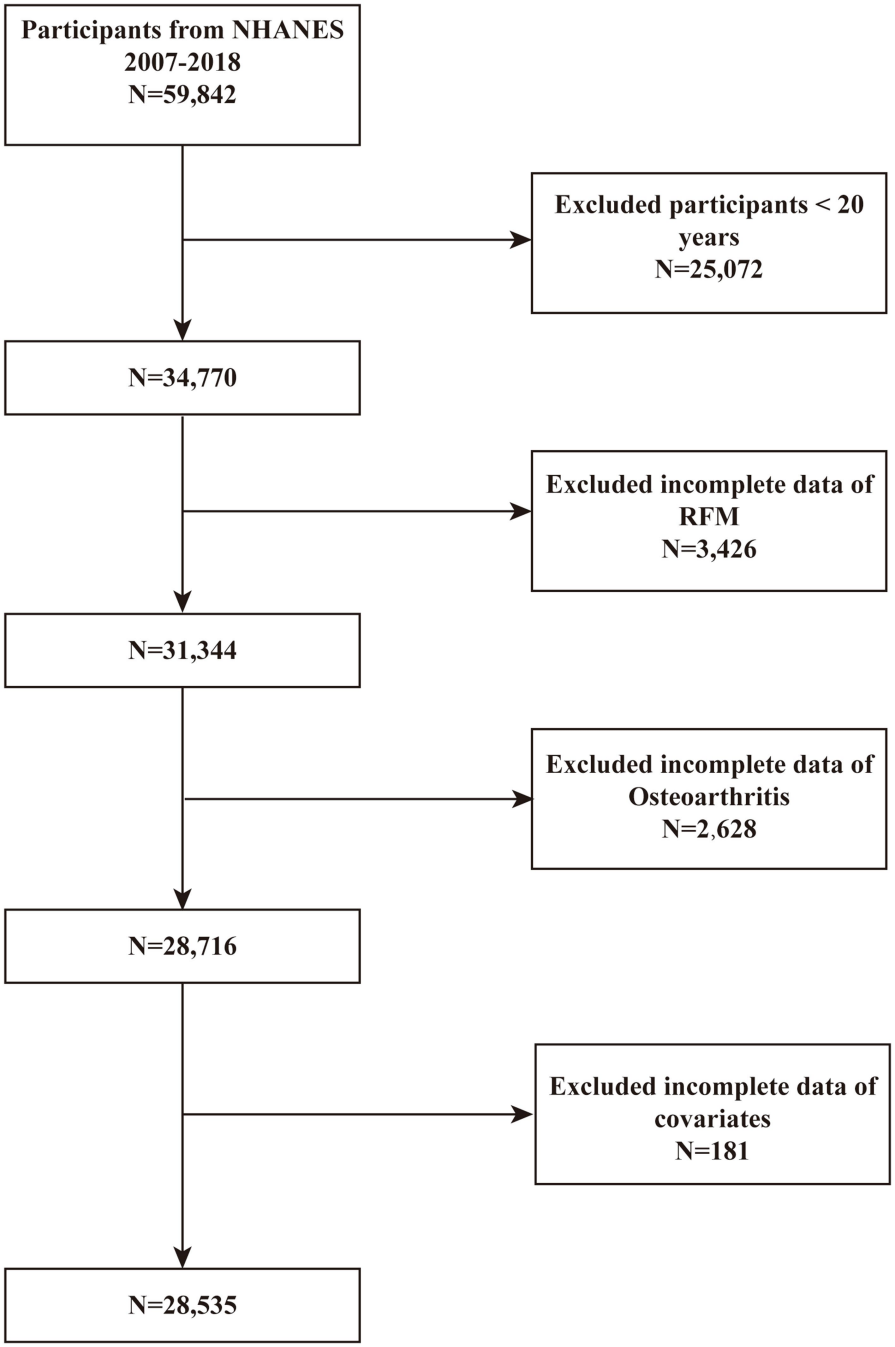
The selection process of subjects from the 2007–2018 NHANES database.

Data were sourced from the NHANES database spanning the years 2007 to 2018, as these cycles contained relevant information concerning obesity and OA. Conducted by the Centers for Disease Control and Prevention (CDC), NHANES is a population-based surveillance system that collects comprehensive health data of individuals in the United States through cross-sectional surveys. All participants provided written informed consent prior to data collection. A total of 59,842 participants were initially recruited for the study, with the exclusion criteria detailed in Figure 1: (1) individuals aged ≤ 20 years (*n* = 25,072); (2) participants lacking complete RFM (*n* = 3,426) and OA data (*n* = 2,628); and (3) participants with incomplete data on covariates, including alcohol consumption, hypertension, BMI, education level, smoking, marital status, cardiovascular disease (CVD), and other variables (*n* = 181). After applying these criteria, 28,535 participants were included in the final analysis.

### Definition of RFM

RFM was calculated using the following formula: RFM = 64 − (20 × Height / Waist Circumference) + (12 × Gender), where gender is coded as 1 for females and 0 for males (17). Height and waist were all available in the database, as measured by trained professionals at the Mobile Examination Center (MEC).

Additionally, we investigated the relationship between WHTR, BMI, WT, and WAIST and cardiorenal syndrome. WHTR = waist (cm)/height(cm). BMI = weight(kg)/height(m)^2^. The body weight (WT) was measured by trained health professionals at the Mobile Examination Center (MEC).

### Definition of OA evaluation

Information on OA diagnosis was collected through the NHANES medical conditions questionnaire. Participants were asked whether a doctor or other healthcare professional had diagnosed them with arthritis. Those who answered “yes” were further classified into OA. If the answer was “No,” the respondents were classified as non-arthritis individuals. Participants who reported “osteoarthritis” were classified as having osteoarthritis. Responses indicating “rheumatoid arthritis” or “other” were considered non-osteoarthritis. Exiting studies have established strong concordance between self-reported OA and clinically diagnostic OA (21).

### Covariates

In our study, several potential covariates were considered to assess their influence on the association between obesity and OA. Covariates in our study comprised demographic variables: age, sex, race, education level and marital status. Health-related variables included diabetes, hypertension, smoking status, drinking status, BMI, CVD, and stroke. Smoking status was classified as no and yes. Drinking status was categorized as no and yes. Specific information on covariates can be found in Supplementary Table 1.

### Statistical analysis

To ensure that the study population is nationally representative, sample-specific weights (WTMEC2YR), stratification (SDMVSTRA), and clustering (SDMVPSU) were incorporated into the analysis. For the combined survey cycles, the two-year weights for each cycle were divided by six to generate new weights for the six-year combined dataset. Continuous variables were summarized using weighted means ± standard deviations, while categorical variables were presented as frequencies and weighted percentages. The evaluation of continuous variables utilized weighted t-tests, whereas weighted chi-square tests were utilized for categorical variables to identify statistical disparities among groups. Furthermore, multivariable logistic regression analysis was conducted to investigate the potential association between RFM and OA. To further investigate this relationship, RFM was stratified into quartiles. The analysis incorporated three distinct models: the crude model (which did not adjust for covariates), a partially adjusted model (Model 1, accounting for age, sex, and race), and a fully adjusted model (Model 2). Subgroup analyses, stratified by age, sex, race, education level, marital status, drinking status, smoking status, diabetes, hypertension, cardiovascular disease and stroke, were conducted to explore the relationship between RFM and OA across different populations. The interaction test in this study was conducted by constructing a Generalized Linear Model (GLM) including interaction terms, which is more appropriate for comparing complex models. Additionally, Receiver Operating Characteristic (ROC) curves were conducted to quantify the predictive validity of obesity indicators for OA, comparing their respective areas under the curve (AUC) values. All statistical analyses in this research were conducted utilizing R (version 4.2.0) and EmpowerStats 4.2.^1^ All statistical analyses used two-tailed tests with statistical significance defined as *p* < 0.05.

## Results

### Baseline characteristics

A total of 28,535 participants were included in this study. The differences in clinical characteristics between the OA and non-OA groups are presented in Table 1. The mean age of the participants was 48.20 years, with 49.07% male and 50.93% female. RFM was significantly higher in OA patients compared to non-OA individuals (39.44 vs. 34.98, *p* < 0.0001). Additionally, sociodemographic factors (age, sex, race, education level, marital status) and health behaviors (smoking status, drinking status), along with BMI were observed substantial between-group differences (*p* < 0.0001). Compared to non-OA individuals, those with OA showed statistically significant increases in prevalence across chronic diseases. (CVD: 16.78% vs. 6.13%; DM: 20.66% vs. 10.75%; hypertension: 60.08% vs. 30.15%; Stroke: 6.70% vs. 2.69%; vigorous physical activity: 13.09% vs. 24.60%; moderate physical activity: 37.48% vs. 41.60%).

**Table 1:** Baseline characteristic of participants.

Characteristics	Total	Non-OA	OA	*P*-value
Participant number		25,341	3,194	-
Age	48.20 ± 17.40	46.33 ± 16.95	63.08 ± 13.24	< 0.0001
BMI (kg/m^2^)	29.00 ± 6.82	28.77 ± 6.69	30.88 ± 7.53	< 0.0001
Race (%)				< 0.0001
Mexican American	4,350 (15.24)	4,089 (16.14)	261 (8.17)	
Other Hispanic	3,020 (10.58)	2,771 (10.93)	249 (7.80)	
Non-Hispanic White	11,484 (40.25)	9,552 (37.69)	1932 (60.49)	
Non-Hispanic Black	6,013 (21.07)	5,506 (21.73)	507 (15.87)	
Other race	3,668 (12.85)	3,423 (13.51)	245 (7.67)	
Sex (%)				< 0.0001
Male	14,003 (49.07)	12,850 (50.71)	1,153 (36.10)	
Female	14,532 (50.93)	12,491 (49.29)	2041 (63.90)	
Education level (%)				< 0.0001
Less than high school	6,670 (23.37)	6,031 (23.80)	639 (20.01)	
High school	6,442 (22.58)	5,717 (22.56)	725 (22.70)	
More than high school	15,423 (54.05)	13,593 (53.64)	1830 (57.29)	
Marital status (%)				0.291
Married/living with partner	17,131 (60.04)	15,241 (60.14)	1890 (59.17)	
Never married/widowed/divorced/separated	11,404 (39.96)	10,100 (39.86)	1,304 (40.83)	
Smoking status (%)				< 0.0001
No	16,247 (56.94)	14,726 (58.11)	1,521 (47.62)	
Yes	12,288 (43.06)	10,615 (41.89)	1,673 (52.38)	
Drinking status (%)				< 0.0001
No	8,102 (28.39)	7,026 (27.73)	1,076 (33.69)	
Yes	20,433 (71.61)	18,315 (72.27)	2,118 (66.31)	
CVD (%)				< 0.0001
No	26,445 (92.68)	23,787 (93.87)	2,658 (83.22)	
Yes	2090 (7.32)	1,554 (6.13)	536 (16.78)	
DM (%)				< 0.0001
No	24,527 (85.95)	22,131 (87.33)	2,396 (75.02)	
Yes	3,383 (11.86)	2,723 (10.75)	660 (20.66)	
Borderline	625 (2.19)	487 (1.92)	138 (4.32)	
Hypertension (%)				< 0.0001
No	18,975 (66.50)	17,700 (69.85)	1,275 (39.92)	
Yes	9,560 (33.50)	7,641 (30.15)	1919 (60.08)	
Stroke (%)				< 0.0001
No	27,639 (96.86)	24,659 (97.31)	2,980 (93.30)	
Yes	896 (3.14)	682 (2.69)	214 (6.70)	
Vigorous physical activity (%)				< 0.0001
No	21,884 (76.69)	19,108 (75.40)	2,776 (86.91)	
Yes	6,651 (23.31)	6,233 (24.60)	418 (13.09)	
Moderate physical activity (%)				< 0.0001
No	16,795 (58.86)	14,798 (58.40)	1997 (62.52)	
Yes	11,740 (41.14)	10,543 (41.60)	1,197 (37.48)	
RFM	35.48 ± 8.72	34.98 ± 8.68	39.44 ± 8.02	< 0.0001

Continuous variables: values are expressed as mean ± standard deviation. Categorical variables: values are expressed as numbers (percentage). RFM, relative fat mass; CVD, cardiovascular disease; OA, osteoarthritis; BMI, body mass index; DM, diabetes mellitus.

### The association between RFM and OA

**Figure 2:**
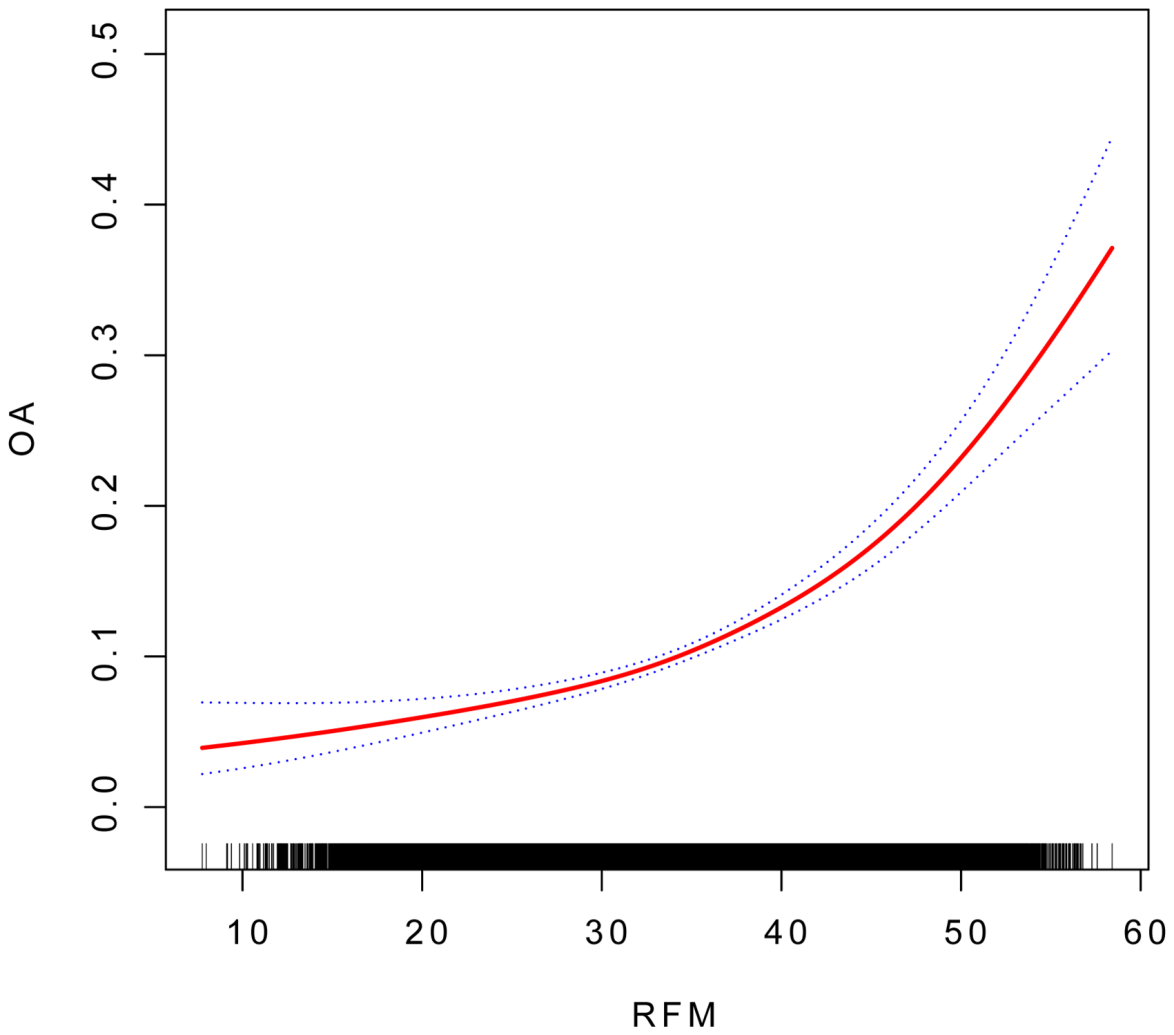
The association between RFM and OA. Smooth curve fitting for the association between RFM and OA. The solid red line represents the smooth curve fit between variables.

Three models to study the association between RFM and OA were used in this analysis: an unadjusted model (Model 1), a model adjusted for core demographics: age, sex and race (Model 2), and a fully adjusted model incorporating disease-related factors based on Model 2 (Model 3). The results from the multivariable logistic regression models (Model 3) are comprehensively presented in Figure 2, demonstrating the association between RF and OA. When RFM was analyzed as a continuous variable, a positive correlation with OA was observed in all models. In Model 3, after adjusting for age, sex, race, education level, marital status, drinking status, smoking status, diabetes, hypertension, cardiovascular disease, and stroke, the association between RFM and OA remained highly significant (OR = 1.05; 95% CI: 1.04–1.07). To further investigate this relationship, RFM was stratified into quartiles. The analysis revealed that in Model 3, participants in the highest RFM quartile demonstrated significantly greater OA prevalence than those in the lowest quartile (OR = 2.51; 95% CI: 1.91–3.31), with a significant trend across quartiles (P for trend < 0.0001) (see Table 2).

**Table 2:** Weighted multivariate logistic regression analysis of RFM and OA.

Exposure	Model 1	Model 2	Model 3
	OR (95%CI)	OR (95%CI)	OR (95%CI)
OA			
RFM (continuous)	1.07 (1.06, 1.07) < 0.0001	1.06 (1.05, 1.08) < 0.0001	1.05 (1.04, 1.07) < 0.0001
RFM (categorical)			
Q1	Reference	Reference	Reference
Q2	1.81 (1.53, 2.15) < 0.0001	1.26 (1.05, 1.52) 0.0162	1.13 (0.93, 1.36) 0.2131
Q3	2.55 (2.16, 3.01) < 0.0001	1.92 (1.52, 2.42) < 0.0001	1.62 (1.27, 2.08) 0.0003
Q4	4.42 (3.81, 5.13) < 0.0001	3.16 (2.46, 4.07) < 0.0001	2.51 (1.91, 3.31) < 0.0001
P for trend	<0.0001	<0.0001	<0.0001

RFM, relative fat mass; Q, quartile; OA, osteoarthritis. Model 1: unadjusted. Model 2: adjusted for age, sex, race. Model 3: adjusted for age, sex, race, education level, marital status, drinking status, smoking status, diabetes, hypertension, cardiovascular disease and stroke.

The threshold effect analysis revealed a statistically significant difference between the linear and piecewise linear regression models (*p* < 0.001). As shown in Table 3, the identified inflection point was 31.67. Below this threshold, the impact of RFM on OA risk was relatively modest, with each unit increase in RFM associated with a 2% increase in risk. However, above this threshold, the effect became substantially stronger, with each unit increase in RFM corresponding to a 7% increase in OA risk.

**Table 3:** Threshold effect analysis of the association of RFM with OA.

Outcome	OA
	OR (95%CI) *P*-value
Model I Fitting by standard linear model	
	1.06 (1.05, 1.07) < 0.0001
Model II Fitting by two-piecewise linear model	
Breakpoint (K)	31.67
< K (OR1)	1.02 (1.00, 1.04) 0.0277
> K (OR2)	1.07 (1.06, 1.08) < 0.0001
OR2/OR1	1.04 (1.02, 1.07) 0.0004
Log likelihood ratio test	<0.001

The threshold effect analysis was adjusted for age, sex, race, education level, marital status, drinking status, smoking status, diabetes, hypertension, cardiovascular disease and stroke.

### Subgroup analysis

To evaluate the robustness of the association between RFM and OA, we conducted subgroup analyses stratified by variables including age group, sex, smoking status, and the presence of hypertension, diabetes, CVD and stroke, with appropriate adjustments made for relevant confounding factors. As evidenced in Table 4, a consistently significant positive association between RFM and OA was observed across all subgroups. The interaction analysis revealed that age and diabetes significantly influenced the relationship between RFM and OA (P for interaction < 0.05). The smoothed fitting curves for other groups can be found in the Supplementary materials.

**Table 4:** Subgroup analysis of the association between RFM and OA.

Subgroup	OA	P for interaction
	OR(95%CI)	
Age group		0.0216
≤60	1.07 (1.05, 1.08) < 0.0001	
>60	1.05 (1.03, 1.06) < 0.0001	
Sex		0.2263
Male	1.05 (1.04, 1.07) < 0.0001	
Female	1.06 (1.05, 1.07) < 0.0001	
Smoking status		0.5025
No	1.06 (1.05, 1.07) < 0.0001	
Yes	1.06 (1.05, 1.07) < 0.0001	
Hypertension		0.1152
No	1.05 (1.04, 1.06) < 0.0001	
Yes	1.07 (1.05, 1.08) < 0.0001	
Diabetes		0.0428
No	1.06 (1.05, 1.07) < 0.0001	
Yes	1.08 (1.05, 1.10) < 0.0001	
Borderline	1.01 (0.97, 1.06) 0.5281	
CVD		0.2289
No	1.06 (1.05, 1.07) < 0.0001	
Yes	1.04 (1.02, 1.07) 0.0003	
Stroke		0.3874
No	1.06 (1.05, 1.07) < 0.0001	
Yes	1.04 (1.00, 1.08) 0.0304	

RFM, relative fat mass; CVD, cardiovascular disease; OA, osteoarthritis. Adjusted for sex, age, educational level, marital status, drinking status, hypertension, diabetes, smoking status, CVD, stroke except the subgroup variable.

**Figure 3:**
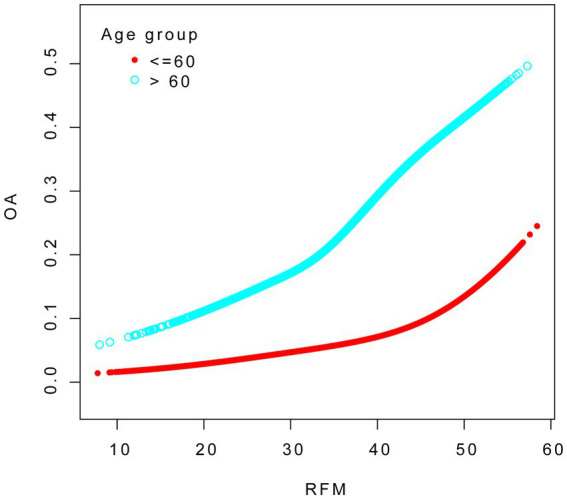
The association between RFM and OA in the strata for age.

We conducted smooth curve fitting to explore the association between RFM and OA across different age groups. The analysis was adjusted for age, sex, race, education level, marital status, drinking status, smoking status, diabetes, hypertension, cardiovascular disease, and stroke (see Figure 3).

**Figure 4:**
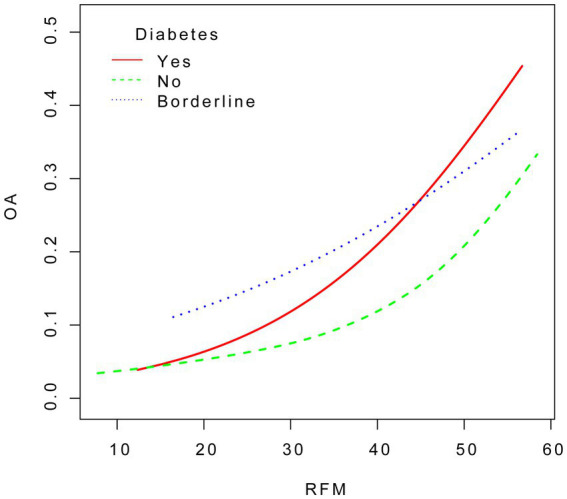
The association between RFM and OA in the strata for diabetes.

We conducted smooth curve fitting to explore the association between RFM and OA across different diabetes groups. The analysis was adjusted for age, sex, race, education level, marital status, drinking status, smoking status, diabetes, hypertension, cardiovascular disease, and stroke (see Figure 4).

### ROC analysis

**Figure 5:**
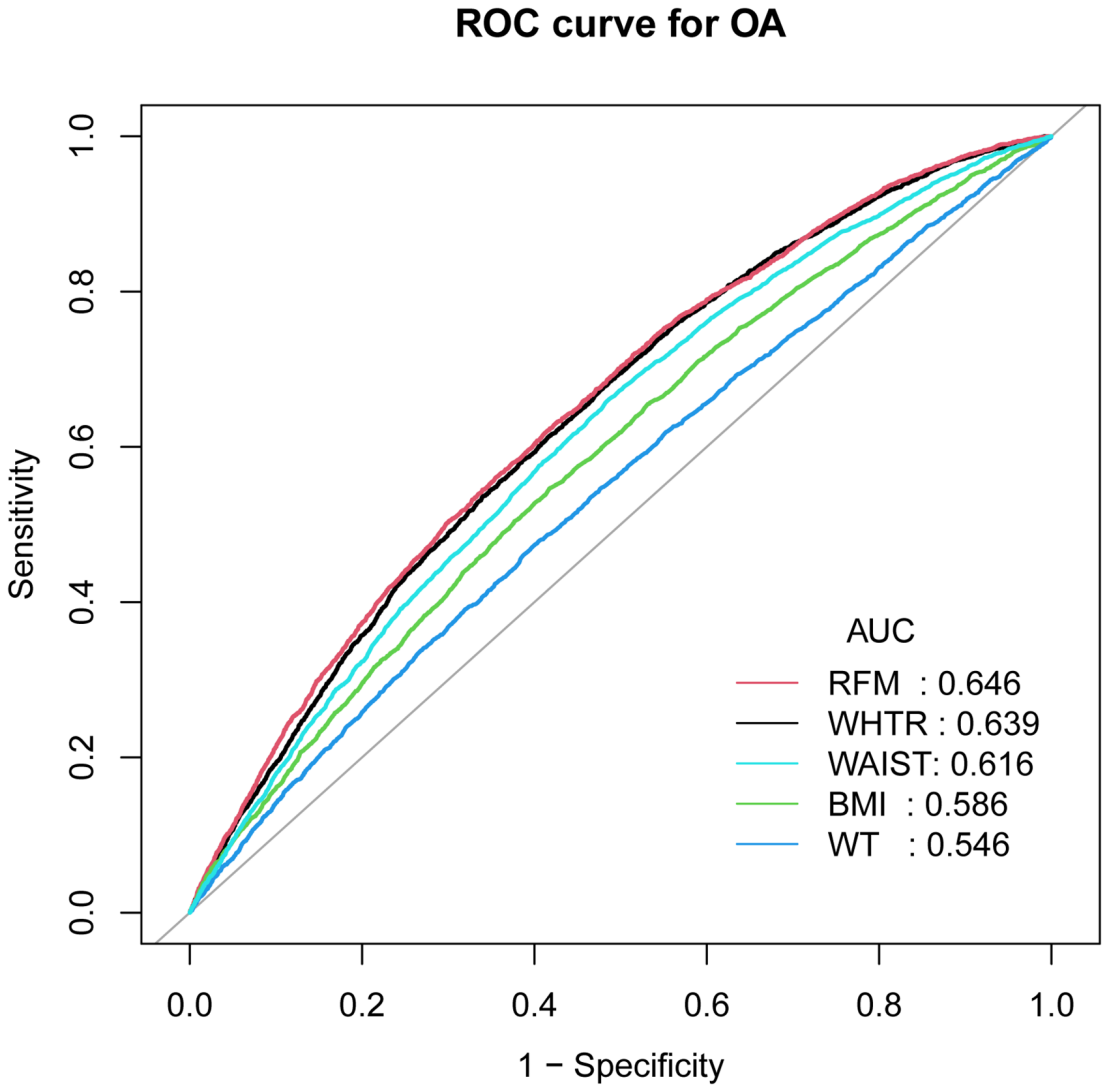
ROC curves and the AUC values of five obesity indicators (RFM, WHTR, WAIST, BMI and WT) in diagnosing OA.

Table 5 demonstrated the diagnostic performance of five different obesity indicators for OA assessment. The results showed that RFM provided superior discriminative ability and accuracy in predicting OA compared to other obesity indicators, including WAIST, BMI, waist-to-height ratio (WTHR), and WT, with area under the curve (AUC) values of 0.646 (see Figure 5).

**Table 5:** The adiposity indicators for predicting OA.

Test	ROC area (AUC)	95%CI low	95%CI up	Best threshold	Specificity	Sensitivity
WHTR	0.6391	0.6292	0.6489	0.5826	0.5129	0.6841
RFM	0.6460	0.6362	0.6558	36.9219	0.5883	0.6177
BMI	0.5863	0.5759	0.5967	28.9850	0.5827	0.5463
WT	0.5460	0.5352	0.5567	82.7500	0.5991	0.4743
WAIST	0.6164	0.6064	0.6265	97.2500	0.5128	0.6619

WHTR, waist-to-height ratio; RFM, relative fat mass; BMI, body mass index; WT, weight; AUC: area under the curve.95% CI: 95% confidence interval.

## Discussion

This study primarily aimed to examine the association between RFM and OA in a nationally representative sample. In this cross-sectional study encompassing 28,535 participants, those with OA exhibited markedly elevated RFM levels compared to non-OA individuals, even after adjusting for confounding factors. Subgroup analyses and interaction tests consistently demonstrated a positive correlation between RFM and OA across all examined subgroups. The ROC analysis further indicated that RFM may serve as a more reliable predictor of OA relative to other anthropometric measures, including WWI, BMI, WT, and WTHR, exhibiting superior discriminative capability for OA evaluation. This study highlights the clinical utility of RFM as a predictive biomarker, highlighting that increased levels of RFM are linked to a higher prevalence of OA.

Given the widespread nature of both obesity and OA, which negatively impact individual health and quality of life while also presenting a considerable public health challenge, understanding this relationship is crucial. Notably, obesity is a well-established risk factor for OA, particularly concerning knee osteoarthritis and hip osteoarthritis. Our findings demonstrated that even after adjusting for the most comprehensive covariates in Model 3, there remained a significant positive association between RFM and the risk of OA, regardless of whether RFM was treated as a continuous or categorical variable. Notably, the OR for the highest RFM quartile (Q4) decreases across models—from 4.42 in Model 1, to 3.16 in Model 2, and to 2.51 in Model 3—suggesting that demographic and metabolic factors may partially mediate the observed association. Previous studies using logistic regression analysis have also confirmed that higher levels of RFM were significantly associated with an increased risk of OA (22). A case–control study involving 400 participants demonstrated a significant positive association between obesity and KOA incidence, with obese participants (BMI ≥ 25 kg/m^2^) exhibiting an elevated disease risk compared to normal-weight (23). Moreover, A population-based cohort study revealed that both overweight and obesity significantly increase the likelihood of developing osteoarthritis in the hands, hips, and knees (24). Notably, KOA exhibited a dose–response relationship with increasing BMI (24). In addition, a cross-sectional survey of Brazil’s obese population revealed a substantially elevated prevalence of self-reported joint pain, as well as hip and knee osteoarthritis (25).

Furthermore, various adiposity metrics beyond BMI including waist circumference (WC) and Weight-Adjusted Waist Index (WWI), demonstrate notable correlations with OA risk. A study conducted among the adult in the United States indicated that higher WWI values were significantly associated with greater OA risk (26). Another study also revealed a significant positive relationship between WWI and OA prevalence, suggesting that higher WWI values were associated with an elevated OA risk (21). A cross-sectional analysis identified a significant relationship between KOA and WC, revealing a particularly pronounced association in males with a WC > 102 cm and females with WC > 88 cm (27). In addition, a comprehensive study in South Korea validated that increased WC was associated with a increased risk of OA (28). RFM appears to represent a more reliable measure for evaluating obesity-OA associations in comparison to conventional metrics. Unlike BMI, RFM provides a more precise evaluation of body fat distribution and has shown a stronger correlation with DXA-measured adiposity indices (17, 29, 30). Moreover, our findings indicate that RFM demonstrates superior predictive performance compared to other obesity indicators, including WHTR, BMI, WT and WAIST. Consistent with our findings, a cross-sectional study involving 81 young Brazilian men also demonstrated that RFM has a stronger association with body fat compared to BMI (30). Similarly, another cross-sectional study showed that RFM demonstrated superior predictive performance for gallstones compared to WHTR, based on area under the ROC curve (AUC) analysis (0.696 VS 0.674) (31). In our study, individuals in Q4 (the highest RFM group) also exhibit a significantly greater risk of developing OA. Given the identified RFM threshold, regular monitoring of RFM may be beneficial from a public health perspective. Early identification of individuals approaching or exceeding this inflection point could facilitate timely interventions, thereby contributing to the prevention of obesity-related conditions such as osteoarthritis and promoting overall health. In addition, patients should be encouraged to engage in regular physical activity, maintain a healthy weight, and strive to keep their RFM within an optimal range, which may help reduce the incidence of OA.

Our study elucidated the complex interplay between obesity and OA. The analysis revealed that each 1-unit increase in RFM was associated with a 5% greater risk of OA. As evident from the RFM formula, waist circumference is the only modifiable variable. Based on the average height for males and females, we estimate that a 1-unit change in RFM corresponds approximately to a 3 cm change in waist circumference for individuals near the risk threshold (waist circumference >102 cm for males and >88 cm for females) (32). For individuals with higher waist circumferences, the same 1-unit change in RFM may correspond to approximately a 4 cm change in waist circumference. These findings suggest that monitoring and managing RFM—particularly through waist circumference control—may serve as an important preventive strategy for OA. Subgroup analyses revealed significant association between RFM and OA, with particularly pronounced variations across age and in participants with versus without diabetes. Age is a well-established risk factor for OA pathogenesis. With advancing age, articular cartilage undergoes progressive dehydration and chondrocyte depletion, contributing to structural degeneration (33). The age-associated chronic low-grade inflammatory state (inflammaging) also exacerbates OA progression through multiple pathways: enhanced antigenic exposure due to cartilage breakdown, accelerated cellular senescence in chondrocytes, and endocrine dysfunction affecting joint homeostasis (34). Moreover, age-related degeneration impacts all articular tissues, with the IFP exhibiting progressive fibrotic transformation and volume reduction (35). Accumulating evidence indicates that metabolic abnormalities induced by diabetes can alter the extracellular matrix components of cartilage cells (36). Moreover, it has been reported that nearly half of patients with DM suffer from various forms of arthritis (37).

Our research incorporates several important strengths. First, the large sample size (*N* = 28,535) significantly enhances the statistical power and reliability of the findings. Second, comprehensive subgroup analyses and adjustment for potential confounding factors improve the validity of the results. Third, RFM offers greater clinical applicability compared to alternative metrics due to its straightforward measurement process. Nevertheless, some limitations should be acknowledged. The cross-sectional design precludes causal inferences regarding the association between RFM and OA. Although we adjusted for multiple confounding variables, residual confounding from unmeasured factors remains possible. Additionally, while the NHANES provides nationally representative data for the United States, the external validity of findings may be constrained when considering populations with different demographic or clinical characteristics.

## Conclusion

RFM was significantly associated with OA in the U. S. population. Compared to other obesity indicators, RFM offers superior practicality due to its ease of measurement, suggesting its potential utility in clinical OA assessment. However, prospective cohort studies are necessary to establish a more definitive causal relationship between RFM and OA.

## Data Availability

The original contributions presented in the study are included in the article/Supplementary material, further inquiries can be directed to the corresponding authors.

## Footnotes

^1^ http://www.empowerstats.com
