# Correction: Association between relative fat mass and osteoarthritis in American adults

**DOI:** 10.3389/fnut.2025.1659839

**Published:** 2025-09-03

**Authors:** Ziyuan Li, Tangchen Yin, Yijing Chen, Jiangsheng Huang, Yuanyue Jiang, Wei Deng

**Affiliations:** ^1^Department of Nursing, School of Health and Nursing, Wuxi Taihu University, Wuxi, China; ^2^Department of Pathology, Kunshan Hospital of Traditional Chinese Medicine, Suzhou, China

**Keywords:** RFM, NHANES, osteoarthritis, obesity, cross-sectional study

In the published article, an error was made with Figure 2 as published. Figure 2 was erroneously replaced with Supplementary Figure 2. The corrected Figure 2 and its caption appear below.

**Figure 2:**
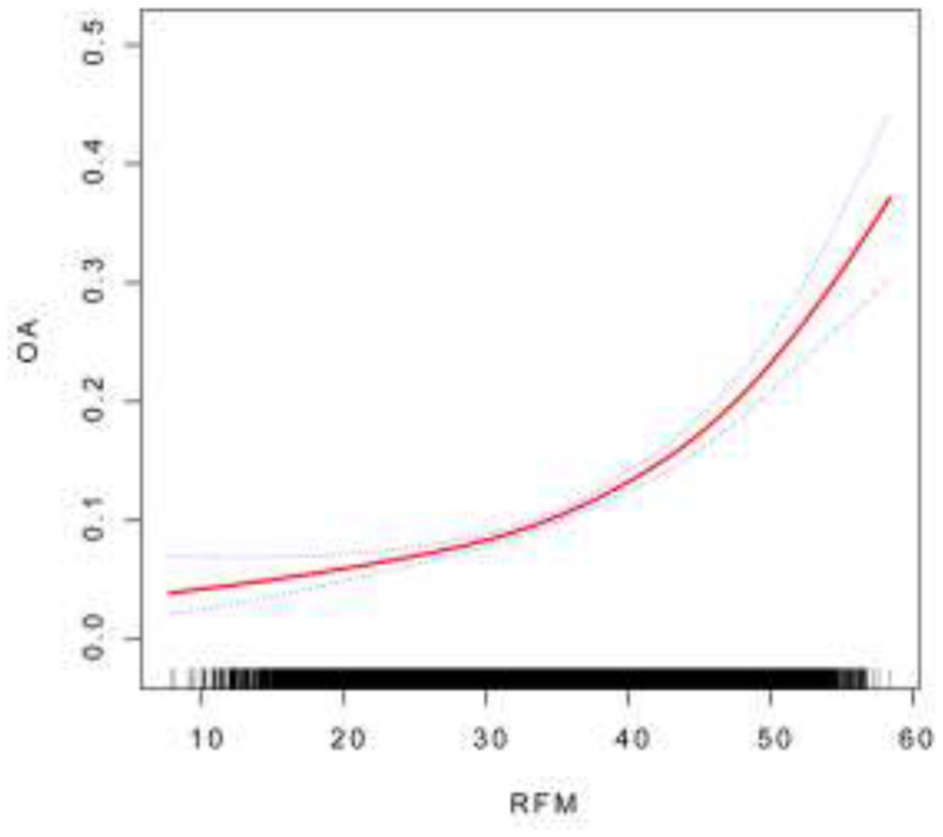
The association between RFM and OA. Smooth curve fitting for the association between RFM and OA. The solid red line represents the smooth curve fit between variables.

In the published article, an error was made with the Supplementary materials. The Supplementary images were omitted from the published article. The correct supplementary images have been updated.

The original article has been updated.

