# Supplementary material for: Association between relative fat mass and osteoarthritis in American adults
Source: Front Nutr. 2025 Jun 18;12:1610950. doi: 10.3389/fnut.2025.1610950 (PMC12213428; doi:10.3389/fnut.2025.1610950)
Supplement: Supplementary file 1 [file Supplementary_file_1.docx]

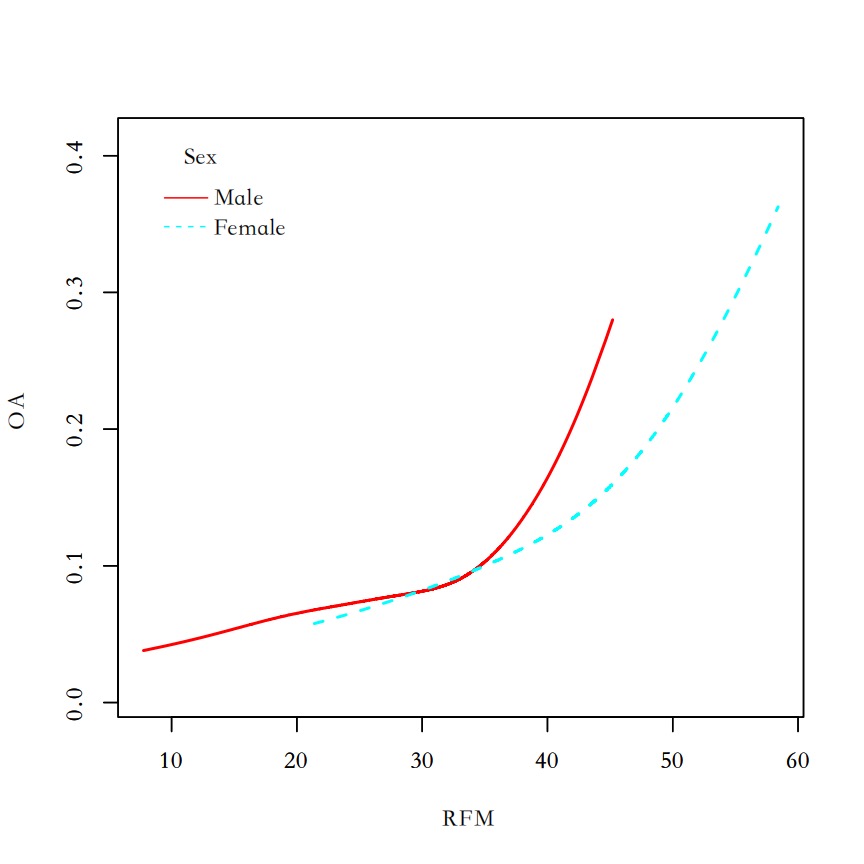


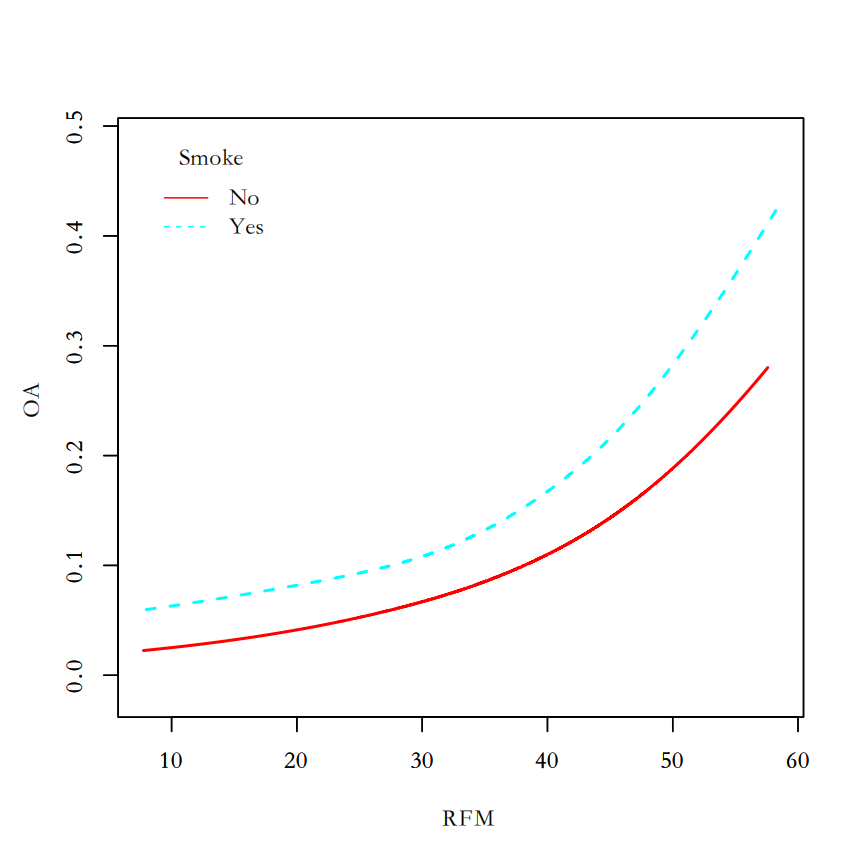


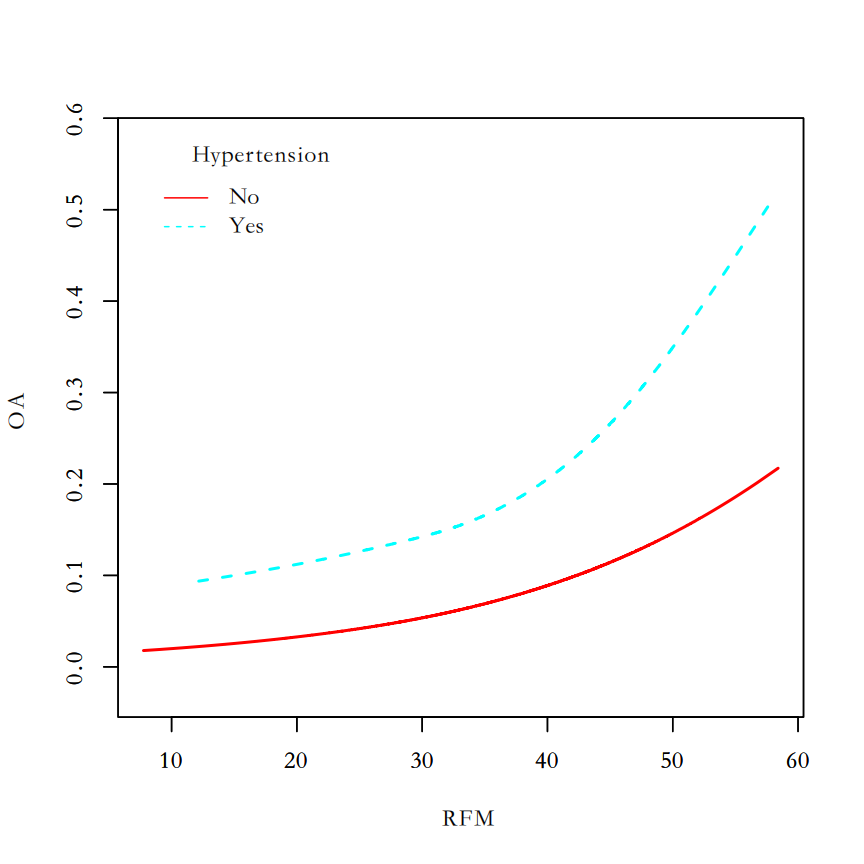


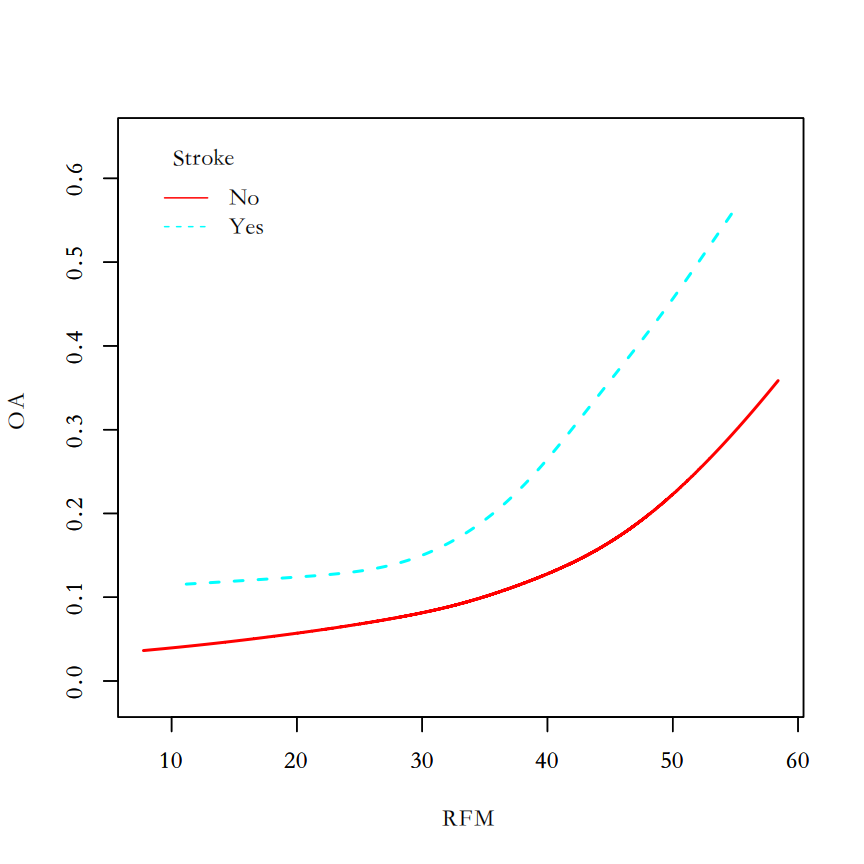


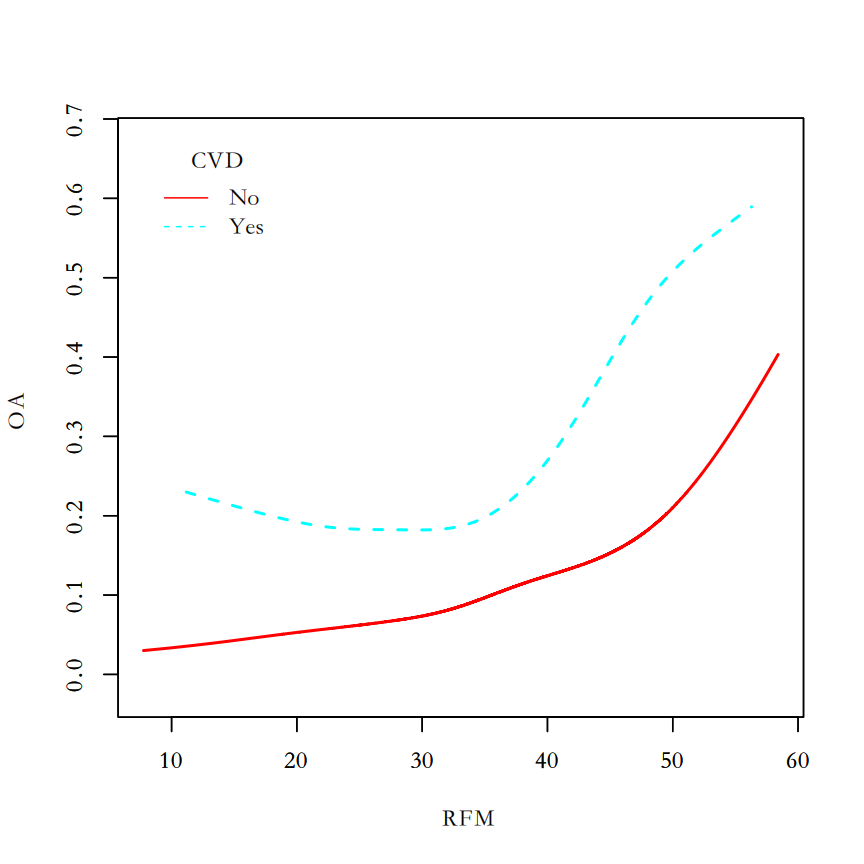


Smooth curve fitting for the association between RFM and different subgroups. Adjusted factors included age, sex, race, education level, marital status, drinking status, smoking status, diabetes, hypertension, cardiovascular disease and stroke except the subgroup variable.
