# Supplementary material for: Association between relative fat mass and osteoarthritis in American adults
Source: Front Nutr. 2025 Jun 18;12:1610950. doi: 10.3389/fnut.2025.1610950 (PMC12213428; doi:10.3389/fnut.2025.1610950)
Supplement: Supplementary file 2 [file Table_1.docx]

**Supplementary table 1. The detailed definition and classification of covariates.**

| **Variables** | **Definitions or Classification** |
| --- | --- |
| Sex | Male, Female. |
| Race | Non-Hispanic White, Non-Hispanic Black, Mexican American, Other Race. |
| Education attainment | High school, less than high school, more than high school. |
| Marital status | Married or living with partner, widowed/divorced/separated/never. |
| Hypertension | Self-reported hypertension. |
| Cardiovascular disease | This section includes inquiries such as “Has a doctor or other health professional ever informed you had congestive heart failure, coronary heart disease, angina (also called angina pectoris), heart attack (also called myocardial infarction), stroke” These questions, labeled as MCQ160B-E in the household questionnaires administered during home interviews, were utilized to identify participants with a history of CVD if they responded “yes” to any of these questions. |
| Stroke | This section includes inquiry such as “Has a doctor or other health professional ever informed you had stroke” his question was utilized to identify participants with a history of stroke if they responded “yes” to this question. |
| BMI (Body Mass Index) | weight (kg)/height^2^ (m^2)^ |
| RFM (Relative Fat Mass) | 64 - (20×height/ waist) + (12×gender) |
| WHtR (Waist-to-Height Ratio) | waist(cm)/ height(cm) |
| Diabetes | Diabetes mellitus was defined by meeting at least one of the following criteria:(1) self-reported diagnosis of diabetes mellitus; (2) use of diabetes medications or insulin |
| Vigorous physical activity | This section includes inquiry such as “{Do you/Does SP} do any vigorous-intensity sports, fitness, or recreational activities that cause large increases in breathing or heart rate like running or basketball for at least 10 minutes continuously” If the participant answered yes, he or she was defined as vigorous physical activity. |
| Moderate physical activity | This section includes inquiry such as “{Do you/Does SP} do any moderate-intensity sports, fitness, or recreational activities that cause a small increase in breathing or heart rate such as brisk walking, bicycling, swimming, or golf for at least 10 minutes continuously?” If the participant answered yes, he or she was defined as moderate physical activity. |
